# 2-Methyl-1,1,3,3-tetra­phenyl­propan-2-ol

**DOI:** 10.1107/S1600536808014761

**Published:** 2008-05-21

**Authors:** Da-Xin Shi, Li-Jun Zhang, Qi Zhang, Jia-Rong Li

**Affiliations:** a School of Chemical Engineering and the Environment, Beijing Institute of Technology, Beijing 100081, People’s Republic of China

## Abstract

The title compound, C_28_H_26_O, was synthesized by condensation of diphenyl­methyl­lithium and ethyl acetate. In one diphenyl­methyl group, the two benzene rings are rotated by 65.0 (3)° with respect to each other, while in the other diphenyl­methyl group, the dihedral angle between the two benzene rings is 84.1 (3)°.

## Related literature

For related literature, see: Bunce & Dowdy (1990 ▶); Ibis & Deniz (2007 ▶); Lednicer *et al.* (1990 ▶).
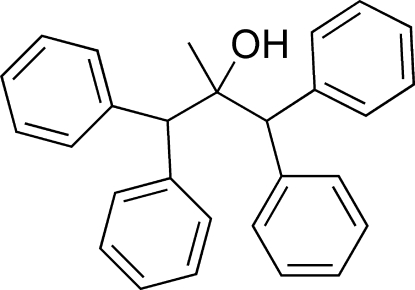


## Experimental

### 

#### Crystal data

C_28_H_26_O
*M* *_r_* = 378.49
Monoclinic, 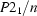

*a* = 8.4313 (4) Å
*b* = 23.8539 (11) Å
*c* = 10.3420 (5) Å
β = 96.624 (3)°
*V* = 2066.09 (17) Å^3^
*Z* = 4
Mo *K*α radiation
μ = 0.07 mm^−1^
*T* = 113 (2) K
0.22 × 0.20 × 0.18 mm

#### Data collection

Rigaku Saturn CCD diffractometer
Absorption correction: multi-scan (*CrystalClear*; Rigaku/MSC, 2005 ▶) *T*_min_ = 0.980, *T*_max_ = 0.985
15409 measured reflections
3633 independent reflections
3442 reflections with *I* > 2σ(*I*)
*R*_int_ = 0.050

#### Refinement

*R*[*F* ^2^ > 2σ(*F* ^2^)] = 0.053
*wR*(*F* ^2^) = 0.116
*S* = 1.11
3633 reflections
267 parameters
H atoms treated by a mixture of independent and constrained refinement
Δρ_max_ = 0.19 e Å^−3^
Δρ_min_ = −0.20 e Å^−3^

### 

Data collection: *CrystalClear* (Rigaku/MSC, 2005 ▶); cell refinement: *CrystalClear*; data reduction: *CrystalClear*; program(s) used to solve structure: *SHELXS97* (Sheldrick, 2008 ▶); program(s) used to refine structure: *SHELXL97* (Sheldrick, 2008 ▶); molecular graphics: *XP* in *SHELXTL* (Sheldrick, 2008 ▶); software used to prepare material for publication: *CrystalStructure* (Rigaku/MSC, 2006 ▶).

## Supplementary Material

Crystal structure: contains datablocks global, I. DOI: 10.1107/S1600536808014761/pk2096sup1.cif

Structure factors: contains datablocks I. DOI: 10.1107/S1600536808014761/pk2096Isup2.hkl

Additional supplementary materials:  crystallographic information; 3D view; checkCIF report

## Figures and Tables

**Fig. 1.**
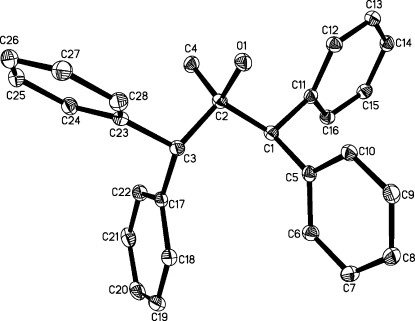
The molecular structure, drawn with 30% probability ellipsoids

**Fig. 2.**
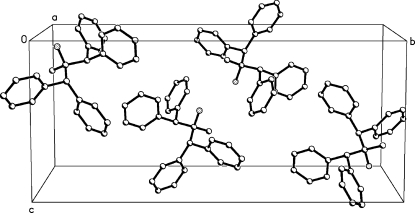
The crystal structure, viewed along a axis

## supplementary crystallographic information

### Comment

Diphenylmethane derivatives are potentially useful precursors to a variety of medicinal agents (Lednicer *et al.*,1990).  In our approach to the preparation of diphenylacetone by reaction of diphenylmethyllithium with ethyl acetate (Bunce & Dowdy, 1990), the title compound, 2-methyl-1,1,3,3-tetraphenylpropan-2-ol, was formed as a double addition product.

There are two diphenymethyl groups in the title compound. In one of these, the two benzene rings are inclined at an angle of 115.0 (3)°, and in the other the dihedral angle between the two benzene rings is 84.1 (3)° which is somewhat different from the corresponding angles found in 3,4,4-trichloro-1-[4-(diphenylmethyl)-piperazine-1-yl]- 2-nitro-1-(propylsulfanyl)-buta-1,3-diene, *viz.* 80.6 (1)° (Ibis & Deniz, 2007).  The plane (C4/C2/O1) and plane (C1/C2/C3) are rotated 92.4 (3)° with respect to each other.

### Experimental

To a stirred solution of ethyl acetate (20 mmol) in dry THF (30 ml) a solution of diphenylmethyllithium (40 mmol) in THF (30 ml) was added dropwise. After stirring at room temperature for 20 min to ensure complete reaction, the mixture was cooled and quenched with HCl (1*M*, 50 ml). The mixture was transferred to a separatory funnel and extrated with ether (40 ml) 3 times. Then, the ether was washed with saturated aqueous Na_2_CO_3_, water and dried with sodium sulfate. Evaporation of the solvent and recrystallization from petroleum ether yielded precipitate as fine colorless needles. *M.p.* 406–408 K; IR (KBr): 3563(O—H), 3026,2938 (C—H) cm^-1^; ^1^H-NMR(CDCl_3_, p.p.m.): 1.30 (3*H*, s), 1.69 (1*H*, s), 4.13 (2*H*, s),7.21–7.45 (20*H*, m); ^13^C-NMR(CDCl_3_, p.p.m.): 25.7, 60.5, 71.4,126.5, 128.2, 130.2, 141.2; The product (100 mg) was dissolved in ethyl acetate (2 ml) and petroleum ether (5 ml) and the solution was kept at room temperature for 5 d yielding colorless single crystals.

### Refinement

All H atoms attached to C atoms were fixed geometrically and treated as riding with C-H = 0.95 Å (C_ar_H), 0.98 Å (RCH_3_) or 1.00 Å (R_3_CH) and U_iso_(H) values of either 1.2 U_eq_ or 1.5 U_eq_ (RCH_3_). The OH hydrogen was found in a difference Fourier map and refined (O-H = 0.90 Å) with Uiso(H) constrained to 1.5 U_eq_ (O).

### Crystal data

**Table:**

C_28_H_26_O	*F*_000_ = 808
*M**_r_* = 378.49	*D*_x_ = 1.217 Mg m^−^^3^
Monoclinic, *P*2_1_/*n*	Mo *K*α radiation λ = 0.71070 Å
Hall symbol: -P 2yn	Cell parameters from 4487 reflections
*a* = 8.4313 (4) Å	θ = 2.2–27.9º
*b* = 23.8539 (11) Å	µ = 0.07 mm^−^^1^
*c* = 10.3420 (5) Å	*T* = 113 (2) K
β = 96.624 (3)º	Prism, colorless
*V* = 2066.09 (17) Å^3^	0.22 × 0.20 × 0.18 mm
*Z* = 4	

### Data collection

**Table:**

Rigaku Saturn CCD diffractometer	3633 independent reflections
Radiation source: rotating anode	3442 reflections with *I* > 2σ(*I*)
Monochromator: confocal multilayer optics	*R*_int_ = 0.050
Detector resolution: 14.63 pixels mm^-1^	θ_max_ = 25.0º
*T* = 113(2) K	θ_min_ = 2.2º
ω scans	*h* = −10→10
Absorption correction: multi-scan(CrystalClear; Rigaku/MSC, 2005)	*k* = −28→28
*T*_min_ = 0.980, *T*_max_ = 0.985	*l* = −12→11
15409 measured reflections	

### Refinement

**Table:**

Refinement on *F*^2^	Hydrogen site location: inferred from neighbouring sites
Least-squares matrix: full	H atoms treated by a mixture of independent and constrained refinement
*R*[*F*^2^ > 2σ(*F*^2^)] = 0.053	*w* = 1/[σ^2^(*F*_o_^2^) + (0.0381*P*)^2^ + 0.9679*P*] where *P* = (*F*_o_^2^ + 2*F*_c_^2^)/3
*wR*(*F*^2^) = 0.116	(Δ/σ)_max_ = 0.001
*S* = 1.11	Δρ_max_ = 0.19 e Å^−^^3^
3633 reflections	Δρ_min_ = −0.20 e Å^−^^3^
267 parameters	Extinction correction: SHELXL97 (Sheldrick, 2008), Fc^*^=kFc[1+0.001xFc^2^λ^3^/sin(2θ)]^-1/4^
Primary atom site location: structure-invariant direct methods	Extinction coefficient: 0.0182 (16)
Secondary atom site location: difference Fourier map	

### Special details

**Table:**

Geometry. All e.s.d.'s (except the e.s.d. in the dihedral angle between two l.s. planes) are estimated using the full covariance matrix. The cell e.s.d.'s are taken into account individually in the estimation of e.s.d.'s in distances, angles and torsion angles; correlations between e.s.d.'s in cell parameters are only used when they are defined by crystal symmetry. An approximate (isotropic) treatment of cell e.s.d.'s is used for estimating e.s.d.'s involving l.s. planes.
Refinement. Refinement of *F*^2^ against ALL reflections. The weighted *R*-factor *wR* and goodness of fit *S* are based on *F*^2^, conventional *R*-factors *R* are based on *F*, with *F* set to zero for negative *F*^2^. The threshold expression of *F*^2^ > σ(*F*^2^) is used only for calculating *R*-factors(gt) *etc*. and is not relevant to the choice of reflections for refinement. *R*-factors based on *F*^2^ are statistically about twice as large as those based on *F*, and *R*- factors based on ALL data will be even larger.

### Fractional atomic coordinates and isotropic or equivalent isotropic displacement parameters (Å^2^)

**Table:**

	*x*	*y*	*z*	*U*_iso_*/*U*_eq_	
O1	0.31548 (15)	0.05491 (5)	0.07175 (11)	0.0266 (3)	
H1	0.261 (3)	0.0232 (9)	0.082 (2)	0.040*	
C1	0.47972 (19)	0.12873 (7)	0.18234 (16)	0.0200 (4)	
H1A	0.5215	0.1406	0.2726	0.024*	
C2	0.3887 (2)	0.07256 (7)	0.19928 (16)	0.0210 (4)	
C3	0.2473 (2)	0.08319 (7)	0.28398 (16)	0.0207 (4)	
H3	0.1715	0.1080	0.2289	0.025*	
C4	0.5084 (2)	0.02774 (7)	0.25298 (18)	0.0257 (4)	
H4A	0.4507	−0.0057	0.2766	0.039*	
H4B	0.5737	0.0423	0.3302	0.039*	
H4C	0.5775	0.0179	0.1865	0.039*	
C5	0.3781 (2)	0.17823 (7)	0.12541 (16)	0.0209 (4)	
C6	0.3655 (2)	0.22646 (7)	0.20063 (17)	0.0242 (4)	
H6	0.4124	0.2268	0.2887	0.029*	
C7	0.2853 (2)	0.27396 (8)	0.14873 (19)	0.0312 (5)	
H7	0.2785	0.3064	0.2011	0.037*	
C8	0.2156 (2)	0.27379 (8)	0.02062 (19)	0.0309 (5)	
H8	0.1609	0.3060	−0.0151	0.037*	
C9	0.2263 (2)	0.22615 (8)	−0.05520 (18)	0.0283 (4)	
H9	0.1781	0.2259	−0.1429	0.034*	
C10	0.3073 (2)	0.17876 (7)	−0.00380 (16)	0.0234 (4)	
H10	0.3145	0.1466	−0.0569	0.028*	
C11	0.6273 (2)	0.12517 (7)	0.10844 (16)	0.0209 (4)	
C12	0.6302 (2)	0.09609 (7)	−0.00935 (16)	0.0247 (4)	
H12	0.5411	0.0739	−0.0425	0.030*	
C13	0.7628 (2)	0.09956 (7)	−0.07790 (17)	0.0258 (4)	
H13	0.7640	0.0792	−0.1567	0.031*	
C14	0.8932 (2)	0.13250 (7)	−0.03209 (17)	0.0259 (4)	
H14	0.9823	0.1352	−0.0802	0.031*	
C15	0.8925 (2)	0.16146 (7)	0.08431 (18)	0.0255 (4)	
H15	0.9812	0.1841	0.1161	0.031*	
C16	0.7610 (2)	0.15716 (7)	0.15446 (17)	0.0236 (4)	
H16	0.7624	0.1764	0.2351	0.028*	
C17	0.2875 (2)	0.11566 (7)	0.41090 (16)	0.0217 (4)	
C18	0.1949 (2)	0.16277 (7)	0.43236 (18)	0.0280 (4)	
H18	0.1090	0.1729	0.3690	0.034*	
C19	0.2261 (3)	0.19528 (8)	0.54486 (19)	0.0348 (5)	
H19	0.1623	0.2273	0.5569	0.042*	
C20	0.3501 (3)	0.18077 (8)	0.63886 (19)	0.0350 (5)	
H20	0.3735	0.2033	0.7143	0.042*	
C21	0.4394 (2)	0.13327 (8)	0.62203 (18)	0.0318 (5)	
H21	0.5215	0.1223	0.6879	0.038*	
C22	0.4095 (2)	0.10121 (8)	0.50852 (16)	0.0261 (4)	
H22	0.4731	0.0691	0.4976	0.031*	
C23	0.1510 (2)	0.02965 (7)	0.30277 (16)	0.0210 (4)	
C24	0.1924 (2)	−0.00805 (7)	0.40487 (17)	0.0258 (4)	
H24	0.2827	−0.0004	0.4661	0.031*	
C25	0.1038 (2)	−0.05649 (8)	0.41854 (18)	0.0294 (4)	
H25	0.1350	−0.0818	0.4878	0.035*	
C26	−0.0301 (2)	−0.06799 (8)	0.33105 (18)	0.0296 (4)	
H26	−0.0910	−0.1009	0.3406	0.035*	
C27	−0.0739 (2)	−0.03107 (8)	0.22989 (19)	0.0287 (4)	
H27	−0.1651	−0.0388	0.1697	0.034*	
C28	0.0153 (2)	0.01748 (7)	0.21599 (17)	0.0252 (4)	
H28	−0.0165	0.0426	0.1466	0.030*	

### Atomic displacement parameters (Å^2^)

**Table:**

	*U*^11^	*U*^22^	*U*^33^	*U*^12^	*U*^13^	*U*^23^
O1	0.0300 (7)	0.0285 (7)	0.0213 (7)	−0.0063 (6)	0.0031 (5)	−0.0030 (5)
C1	0.0198 (9)	0.0214 (9)	0.0188 (8)	−0.0009 (7)	0.0027 (7)	−0.0011 (7)
C2	0.0238 (9)	0.0214 (9)	0.0177 (8)	0.0001 (7)	0.0021 (7)	−0.0016 (7)
C3	0.0212 (9)	0.0208 (9)	0.0205 (9)	0.0019 (7)	0.0037 (7)	0.0013 (7)
C4	0.0252 (10)	0.0236 (9)	0.0290 (10)	0.0027 (7)	0.0056 (8)	0.0040 (7)
C5	0.0180 (9)	0.0234 (9)	0.0221 (9)	−0.0016 (7)	0.0057 (7)	0.0019 (7)
C6	0.0245 (9)	0.0240 (9)	0.0245 (9)	−0.0012 (7)	0.0041 (7)	−0.0024 (7)
C7	0.0361 (11)	0.0231 (10)	0.0362 (11)	0.0036 (8)	0.0116 (9)	−0.0011 (8)
C8	0.0329 (11)	0.0273 (10)	0.0339 (11)	0.0088 (8)	0.0104 (8)	0.0071 (8)
C9	0.0274 (10)	0.0332 (11)	0.0250 (10)	0.0058 (8)	0.0064 (8)	0.0054 (8)
C10	0.0239 (9)	0.0246 (9)	0.0224 (9)	0.0033 (7)	0.0058 (7)	0.0011 (7)
C11	0.0215 (9)	0.0196 (9)	0.0217 (9)	0.0031 (7)	0.0027 (7)	0.0034 (7)
C12	0.0244 (9)	0.0252 (9)	0.0248 (9)	−0.0015 (7)	0.0037 (7)	−0.0022 (7)
C13	0.0274 (10)	0.0260 (10)	0.0244 (9)	0.0017 (8)	0.0052 (8)	−0.0009 (7)
C14	0.0234 (9)	0.0264 (10)	0.0292 (10)	0.0017 (7)	0.0089 (8)	0.0041 (8)
C15	0.0217 (9)	0.0227 (9)	0.0322 (10)	−0.0014 (7)	0.0032 (8)	−0.0001 (7)
C16	0.0237 (9)	0.0238 (9)	0.0231 (9)	0.0007 (7)	0.0017 (7)	−0.0011 (7)
C17	0.0240 (9)	0.0215 (9)	0.0210 (9)	−0.0042 (7)	0.0081 (7)	0.0002 (7)
C18	0.0326 (11)	0.0239 (10)	0.0294 (10)	−0.0018 (8)	0.0113 (8)	0.0005 (8)
C19	0.0460 (13)	0.0260 (10)	0.0362 (11)	−0.0046 (9)	0.0212 (10)	−0.0065 (8)
C20	0.0457 (12)	0.0347 (11)	0.0272 (10)	−0.0174 (9)	0.0155 (9)	−0.0111 (8)
C21	0.0343 (11)	0.0390 (11)	0.0224 (9)	−0.0122 (9)	0.0044 (8)	−0.0006 (8)
C22	0.0287 (10)	0.0262 (10)	0.0242 (9)	−0.0040 (8)	0.0057 (8)	−0.0011 (7)
C23	0.0224 (9)	0.0196 (9)	0.0219 (9)	0.0017 (7)	0.0060 (7)	−0.0017 (7)
C24	0.0279 (10)	0.0277 (10)	0.0222 (9)	−0.0009 (8)	0.0045 (7)	0.0005 (7)
C25	0.0358 (11)	0.0264 (10)	0.0277 (10)	−0.0007 (8)	0.0105 (8)	0.0035 (8)
C26	0.0295 (10)	0.0269 (10)	0.0347 (11)	−0.0051 (8)	0.0139 (8)	−0.0030 (8)
C27	0.0232 (10)	0.0292 (10)	0.0342 (10)	−0.0040 (8)	0.0052 (8)	−0.0049 (8)
C28	0.0237 (9)	0.0256 (9)	0.0263 (10)	0.0024 (7)	0.0032 (7)	0.0013 (7)

### Geometric parameters (Å, °)

**Table:**

O1—C2	1.453 (2)	C13—C14	1.389 (3)
O1—H1	0.90 (2)	C13—H13	0.9500
C1—C5	1.536 (2)	C14—C15	1.388 (3)
C1—C11	1.536 (2)	C14—H14	0.9500
C1—C2	1.564 (2)	C15—C16	1.397 (2)
C1—H1A	1.0000	C15—H15	0.9500
C2—C4	1.530 (2)	C16—H16	0.9500
C2—C3	1.579 (2)	C17—C22	1.399 (2)
C3—C17	1.528 (2)	C17—C18	1.400 (2)
C3—C23	1.538 (2)	C18—C19	1.398 (3)
C3—H3	1.0000	C18—H18	0.9500
C4—H4A	0.9800	C19—C20	1.387 (3)
C4—H4B	0.9800	C19—H19	0.9500
C4—H4C	0.9800	C20—C21	1.383 (3)
C5—C10	1.399 (2)	C20—H20	0.9500
C5—C6	1.400 (2)	C21—C22	1.399 (3)
C6—C7	1.394 (3)	C21—H21	0.9500
C6—H6	0.9500	C22—H22	0.9500
C7—C8	1.386 (3)	C23—C24	1.400 (2)
C7—H7	0.9500	C23—C28	1.401 (2)
C8—C9	1.390 (3)	C24—C25	1.392 (3)
C8—H8	0.9500	C24—H24	0.9500
C9—C10	1.394 (2)	C25—C26	1.390 (3)
C9—H9	0.9500	C25—H25	0.9500
C10—H10	0.9500	C26—C27	1.385 (3)
C11—C16	1.398 (2)	C26—H26	0.9500
C11—C12	1.405 (2)	C27—C28	1.398 (2)
C12—C13	1.394 (2)	C27—H27	0.9500
C12—H12	0.9500	C28—H28	0.9500
			
C2—O1—H1	108.2 (13)	C14—C13—C12	120.65 (16)
C5—C1—C11	107.46 (13)	C14—C13—H13	119.7
C5—C1—C2	116.24 (14)	C12—C13—H13	119.7
C11—C1—C2	116.40 (13)	C15—C14—C13	119.67 (16)
C5—C1—H1A	105.2	C15—C14—H14	120.2
C11—C1—H1A	105.2	C13—C14—H14	120.2
C2—C1—H1A	105.2	C14—C15—C16	119.77 (17)
O1—C2—C4	108.69 (13)	C14—C15—H15	120.1
O1—C2—C1	108.02 (13)	C16—C15—H15	120.1
C4—C2—C1	109.12 (14)	C15—C16—C11	121.34 (16)
O1—C2—C3	106.33 (13)	C15—C16—H16	119.3
C4—C2—C3	114.80 (13)	C11—C16—H16	119.3
C1—C2—C3	109.65 (13)	C22—C17—C18	117.40 (16)
C17—C3—C23	112.37 (13)	C22—C17—C3	124.53 (16)
C17—C3—C2	116.80 (14)	C18—C17—C3	118.06 (16)
C23—C3—C2	112.33 (13)	C19—C18—C17	121.51 (18)
C17—C3—H3	104.6	C19—C18—H18	119.2
C23—C3—H3	104.6	C17—C18—H18	119.2
C2—C3—H3	104.6	C20—C19—C18	119.93 (18)
C2—C4—H4A	109.5	C20—C19—H19	120.0
C2—C4—H4B	109.5	C18—C19—H19	120.0
H4A—C4—H4B	109.5	C21—C20—C19	119.59 (18)
C2—C4—H4C	109.5	C21—C20—H20	120.2
H4A—C4—H4C	109.5	C19—C20—H20	120.2
H4B—C4—H4C	109.5	C20—C21—C22	120.35 (19)
C10—C5—C6	118.16 (16)	C20—C21—H21	119.8
C10—C5—C1	122.05 (15)	C22—C21—H21	119.8
C6—C5—C1	119.58 (15)	C17—C22—C21	121.15 (18)
C7—C6—C5	121.22 (17)	C17—C22—H22	119.4
C7—C6—H6	119.4	C21—C22—H22	119.4
C5—C6—H6	119.4	C24—C23—C28	117.65 (16)
C8—C7—C6	119.91 (17)	C24—C23—C3	122.67 (15)
C8—C7—H7	120.0	C28—C23—C3	119.68 (15)
C6—C7—H7	120.0	C25—C24—C23	121.34 (17)
C7—C8—C9	119.59 (17)	C25—C24—H24	119.3
C7—C8—H8	120.2	C23—C24—H24	119.3
C9—C8—H8	120.2	C26—C25—C24	120.16 (17)
C8—C9—C10	120.59 (17)	C26—C25—H25	119.9
C8—C9—H9	119.7	C24—C25—H25	119.9
C10—C9—H9	119.7	C27—C26—C25	119.49 (17)
C9—C10—C5	120.51 (16)	C27—C26—H26	120.3
C9—C10—H10	119.7	C25—C26—H26	120.3
C5—C10—H10	119.7	C26—C27—C28	120.31 (18)
C16—C11—C12	118.10 (16)	C26—C27—H27	119.8
C16—C11—C1	117.90 (15)	C28—C27—H27	119.8
C12—C11—C1	123.70 (15)	C27—C28—C23	121.03 (17)
C13—C12—C11	120.46 (16)	C27—C28—H28	119.5
C13—C12—H12	119.8	C23—C28—H28	119.5
C11—C12—H12	119.8		
			
C5—C1—C2—O1	60.36 (17)	C11—C12—C13—C14	−1.0 (3)
C11—C1—C2—O1	−67.83 (17)	C12—C13—C14—C15	1.1 (3)
C5—C1—C2—C4	178.36 (13)	C13—C14—C15—C16	0.1 (3)
C11—C1—C2—C4	50.16 (19)	C14—C15—C16—C11	−1.5 (3)
C5—C1—C2—C3	−55.11 (18)	C12—C11—C16—C15	1.6 (3)
C11—C1—C2—C3	176.70 (13)	C1—C11—C16—C15	−172.30 (15)
O1—C2—C3—C17	−165.43 (13)	C23—C3—C17—C22	78.1 (2)
C4—C2—C3—C17	74.36 (19)	C2—C3—C17—C22	−53.8 (2)
C1—C2—C3—C17	−48.89 (19)	C23—C3—C17—C18	−101.00 (18)
O1—C2—C3—C23	62.63 (17)	C2—C3—C17—C18	127.07 (16)
C4—C2—C3—C23	−57.59 (19)	C22—C17—C18—C19	2.0 (3)
C1—C2—C3—C23	179.17 (13)	C3—C17—C18—C19	−178.79 (16)
C11—C1—C5—C10	64.1 (2)	C17—C18—C19—C20	−0.6 (3)
C2—C1—C5—C10	−68.3 (2)	C18—C19—C20—C21	−1.7 (3)
C11—C1—C5—C6	−110.57 (17)	C19—C20—C21—C22	2.6 (3)
C2—C1—C5—C6	116.99 (17)	C18—C17—C22—C21	−1.1 (2)
C10—C5—C6—C7	−0.3 (3)	C3—C17—C22—C21	179.75 (16)
C1—C5—C6—C7	174.63 (16)	C20—C21—C22—C17	−1.2 (3)
C5—C6—C7—C8	0.4 (3)	C17—C3—C23—C24	−46.2 (2)
C6—C7—C8—C9	0.0 (3)	C2—C3—C23—C24	87.94 (19)
C7—C8—C9—C10	−0.4 (3)	C17—C3—C23—C28	133.29 (16)
C8—C9—C10—C5	0.5 (3)	C2—C3—C23—C28	−92.59 (18)
C6—C5—C10—C9	−0.2 (2)	C28—C23—C24—C25	1.2 (3)
C1—C5—C10—C9	−174.93 (15)	C3—C23—C24—C25	−179.32 (16)
C5—C1—C11—C16	87.47 (18)	C23—C24—C25—C26	−0.9 (3)
C2—C1—C11—C16	−140.18 (16)	C24—C25—C26—C27	0.4 (3)
C5—C1—C11—C12	−86.06 (19)	C25—C26—C27—C28	−0.2 (3)
C2—C1—C11—C12	46.3 (2)	C26—C27—C28—C23	0.5 (3)
C16—C11—C12—C13	−0.3 (3)	C24—C23—C28—C27	−1.0 (2)
C1—C11—C12—C13	173.18 (16)	C3—C23—C28—C27	179.51 (15)
